# Face Thermal Map of the Mexican Population in the Basal State

**DOI:** 10.3390/ijerph192114208

**Published:** 2022-10-31

**Authors:** Daniel Jaramillo-Quintanar, Irving A. Cruz-Albarran, Benjamin Dominguez-Trejo, David A. Rodriguez-Medina, Luis Alberto Morales-Hernandez

**Affiliations:** 1 Mechatronics/Engineering Faculty, Campus San Juan del Rio, Autonomous University of Queretaro, San Juan del Rio 76807, Queretaro, Mexico; 2 Postgraduate Studies Division, Psychology Faculty, National Autonomous University of Mexico, Mexico City 04510, Mexico; 3 Department of Sociology, Division of Social Sciences and Humanities, Universidad Autónoma Metropolitana, Mexico City 52919, Mexico

**Keywords:** thermal map, basal state, face, region of interest, Mexican population

## Abstract

There has been a wide use of thermal images of the human body in recent years, specifically images with thermal information of regions of interest (ROI) in the face; this information can be used for epidemiological, clinical, and/or psychological purposes. Due to this, it is important to have plenty of information on temperature in these ROIs in the basal state that allows their use as a reference in terms of their thermal analysis. In this work, a face thermal map of the Mexican population in the basal state (*n* = 196) is created, adding the comparison between different population groups, such as gender, age, and clinical status, obtaining results of great interest for future research. The *t*-test for independent samples was applied to the ROIs with normal distribution and Mann–Whitney u-test to the ones that did not present normal distribution. Statistically significant differences were found in some of the ROI comparisons like the corrugator, the supraorbitals, and the chin between the control and clinical groups, as well as in the differentiation by age (*p* < 0.05).

## 1. Introduction

Over the years, different tools have been developed to facilitate the identification, the evaluation, and the classification of the problems that arise in human beings, trying to understand the operation of the body, its reactions [1], and its signals [2]. Some of the changes presented in the body that make evident an alteration to normality are the increases and the decreases of temperature in specific areas [3,4]. It has been determined that thermal changes in specific body areas can represent numerous things, such as the body’s response to fatigue [5], inflammatory processes caused by infections [6] and diseases [7], or emotional changes [8].

Temperature changes in distinct areas of the face can be caused by different factors, such as environmental, individual, and technical factors [9]; these thermal changes are also related to emotions or changes in these, due to variations in peripheral blood supply as part of the homeostasis process [10]. Accordingly, it is important to generate databases that contain reliable information on the temperature in the most outstanding regions of interest (ROI), such as the nose, the forehead [11], the supraorbital [12], and the cheeks [13] among others.

There are large amounts of human image databases or maps, oriented to different purposes and with distinct characteristics, such as body image databases [14], the feet [15] or the brain [16], and even thermal images. Within the human thermal image databases, there are some to be used for facial recognition [17]; some even combine thermal images with visible images to give greater recognition strengths [18,19,20], and others add annotations, such as [21,22]. Research that incorporates both images with the aim of expression recognition using facial action units (AUs) and temperature can be found [23,24]; the facial AUs are used for emotion recognition as well as the infrared images, but AUs are focused in a physical change while the ROIs in thermal images look for a physiological variation. There are also some databases that show thermal human body maps in different population groups [25,26], or comparison between genders [27,28], but a database that can be used as a reference when looking for a thermal profile of the human face in the basal state (A resting metabolic state early in the morning after a minimum of 12 h of fasting [29]) under controlled conditions for epidemiological, clinical, and/or psychological purposes is not available to researchers.

In this work, a thermal map of the face in the basal state of the Mexican population (*n* = 196) under controlled conditions was created, with the evaluation of eight ROIs: forehead, corrugator muscle, right supraorbital, left supraorbital, nose, right masseter, left masseter and chin, incorporating the comparison between genders, ages, and, a clinical group against a control group. The data obtained in this work could be used as a reference to compare temperatures in the ROIs addressed, oriented mainly to emotional changes but can be used for the purposes that researchers require.

## 2. Materials and Methods

The study was conducted following the General Health Law and in accordance with the Helsinki statement, the research project was submitted to the bioethics committee for research of the engineering faculty of the Autonomous University of Querétaro (UAQ) with a registration key “CEAIFI-132-2019-TP”. All participants signed an informed consent letter explaining the possibility of abandoning the study if they decided so.

### 2.1. Technological Equipment

For the acquisition of thermal images, a FLIR A310 camera was used, with thermal sensitivity of 0.05 at 30 °C, an infrared resolution of 320 × 240 pixels, and a spectral range between 7.5 and 13 µm. This camera was installed on a tripod at a height of 1.2 m and a distance of 1.2 m from the participant. The skin emissivity was set to 0.98 according to previous studies [30]. To measure environmental conditions, a fluke 975 equipment was used to obtain air quality. Additionally, to measure the reflected apparent temperature, a fluke laser thermometer was used.

### 2.2. Conditioned Space

**Figure 1:**
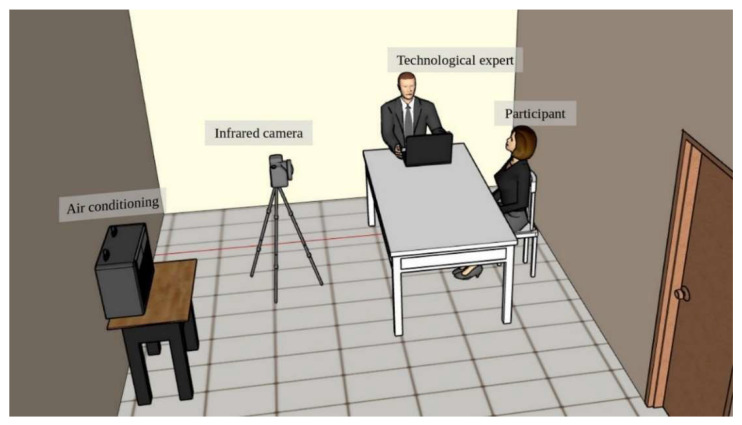
Conditioned space.

To accomplish the study, a controlled environment for the acquisition of the thermal images was created [10] inside a room measuring 2.5 m long, 3 m wide, and 2.5 m high, with a temperature of 20 ± 2 °C, constant lighting, without external incidents of light, and relative humidity of 45 to 60%. To achieve this goal an air conditioning system was used (Figure 1).

The participants were invited one by one to the conditioned room; they were instructed to make themselves comfortable on the chair provided and an acclimatization time of 10 min to regulate their temperature was given to obtain the thermal image of their face in the basal state.

### 2.3. Database

The study of where the images were collected for the database was carried out over several years, from 2016 to 2020 different image acquisitions were made at the Autonomous University of Querétaro “UAQ”, the University of Guadalajara “UDG” and the National Autonomous University of Mexico “UNAM”. Collecting images of 196 participants in the period from March 2016 to November 2018. All participants signed an informed consent letter previously reviewed by the aforementioned bioethics committee in conjunction with the research project.

#### 2.3.1. Regions of Interest (ROI)

**Figure 2:**
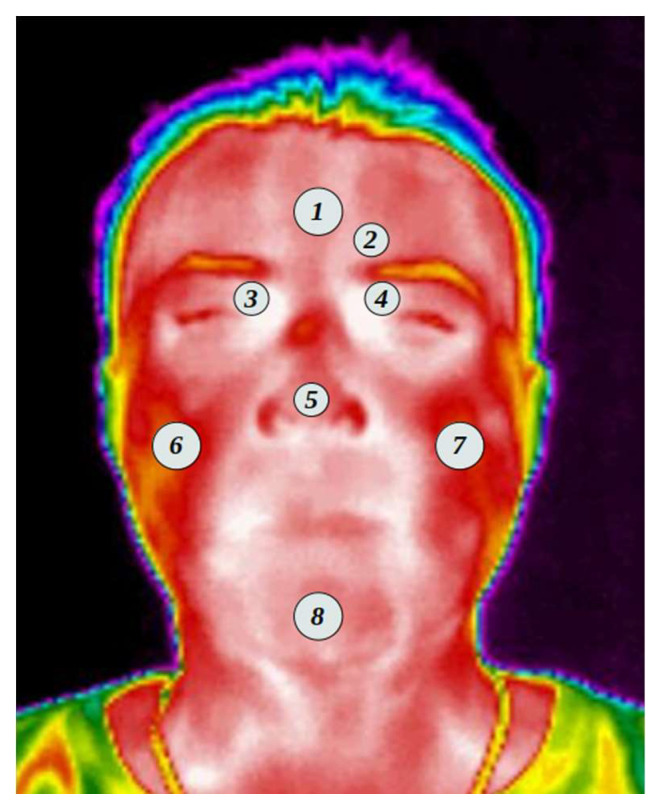
Regions of interest ROI.

A thermal map of the face was created for each of the participants, taking into consideration the ROI according to previous studies [8,31,32,33]. Figure 2 shows the regions where the temperature was evaluated.

The following list shows the ROI selected and shown in Figure 2. From which an average of the values found in the area within the selected region was obtained.

1. Forehead;
2. Corrugator muscle;
3. Right supraorbital;
4. Left supraorbital;
5. Nose;
6. Right masseter;
7. Left masseter;
8. Chin.

The aforementioned ROIs were carefully selected according to what was found in previous studies and based on what was described by [10]. The selected points are equivalent to those of the surface electromyographic psychophysiological recording: 1, 2, and 8 correspond to the areas of reactivity to stress; Items 3, 4, and 5 have been linked to cognitive and affective autonomic responses; while items 6 and 7 concern positive emotional responses. The frontal muscles, the masseter, and the chin muscles require increased blood supply (when the long muscles are activated) [34] and the corrugator muscle responses have been associated with emotions [32,33].

The ROIs were set to circles where the 2, 3, 4, and 5 ROIs were set to a radius of 4 pixels, while the 1, 6, 7, and 8 were to a radius of 5 pixels due to the area needed to cover in each section. Every one of the ROIs was carefully selected by hand to have an appropriate position.

#### 2.3.2. Thermal Evaluation

To obtain the average temperature of each ROI, a thermal matrix of the images is created, using Equation (1) proposed by [35].

(1) $$T_{r}=T_{min}+\left(\frac{T_{gray}}{T_{mgv}}\left(T_{max}-T_{min}\right)\right)$$

where *T_r_* is the thermal value of the pixel selected in the thermogram, *T_max_* and *T_min_* are the minimum and maximum temperature values in °C, *T_gray_* represents the grayscale value of the pixel and *T_mgv_* is the largest grayscale value within the thermogram.

By having the thermal matrix, the ROIs were selected and the average temperature within each area was calculated.

#### 2.3.3. Exclusion Criteria

To have a clean and useful database, a strict series of exclusion criteria were applied, to leave only the images of the face of people who did not present any obstruction for taking the image. Within the exclusion criteria are the use of glasses, either sunglasses or magnifying glasses, beard, hair on the forehead, or any possible object that is obstructing getting the thermal image.

It was also sent a list of instructions to the participants before the study, asking them not to use cosmetics, creams or deodorants for that day and with a minimum of 12 h of fasting at the time of the study.

A general data questionnaire was carried out, questions were asked about activities, substances, diet, health status, medication intake, and time of the menstrual cycle (in the case of women). Due to the nature of the study, only in the case of the clinical group (under medical treatment) was medication intake not restricted (which could have an effect on autonomic activity); that is, the participants continued their medical regimen so as not to interrupt their treatment. This should be taken into consideration for future comparison of psychophysiological processes in patients with disease (chronic or acute).

## 3. Results

A total of 196 thermal images were collected, a number that was reduced after applying the exclusion criteria described in Section 2.3.3. Having a final sample of 152. Table 1 shows the most outstanding data from the database.

### 3.1. Thermal Evaluation

The average temperature values of each ROI described in Section 2.3.1 were obtained. Table 2 shows the average temperatures contained in the database, as well as the *p*-value obtained by applying the Kolmogorov–Smirnov normality test.

**Figure 3:**
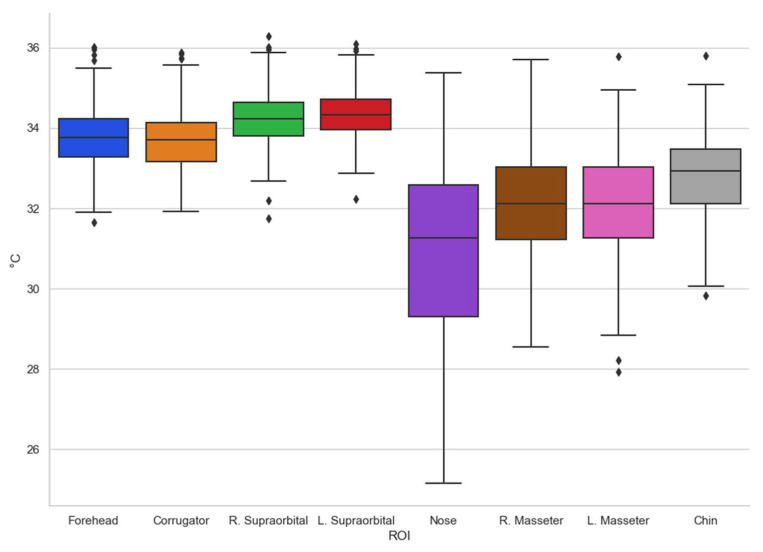
Box-and-whisker chart (all).

To have a more suitable appreciation of how the data obtained behave, the box-and-whisker plot of the entire database obtained is shown (Figure 3).

According to what is shown in Figure 3, the trends can be seen in terms of the temperature shown in the different regions of interest evaluated, the one with the greatest variation being the nose, which is one of the most studied and in which other authors have already found that they present consistent results [8,36]. It is interesting to remark that temperatures in other regions of interest are clustered with minor variations as in the case of supraorbitals.

By having the general temperature data of the regions of interest, some comparisons were made to highlight some possible causal factors obtained on the different ways in which the thermal map of the face behaves in specific population groups. In this section, a discussion will be made about the most outstanding differences found when comparing temperature according to age, gender, and a comparison between a control group and a clinical group.

### 3.2. Gender Comparison

One of the comparisons that comes to mind when it is necessary to identify differences between two different groups within a population is the one made by gender, since it is well known that there are clear differences according to this. Table 3 summarizes the contrasts of temperature in the regions of interest by gender (male, 48; female, 47) as well as the *p*-values of the statistical test *t*-test for independent samples in the case of the regions with normal distribution and the Mann–Whitney u-test for those that did not present normal distribution. Finally, the value of the effect size “r” is presented. The gender comparison was made only with the control group to avoid possible alterations due to imbalance between groups.

**Figure 4:**
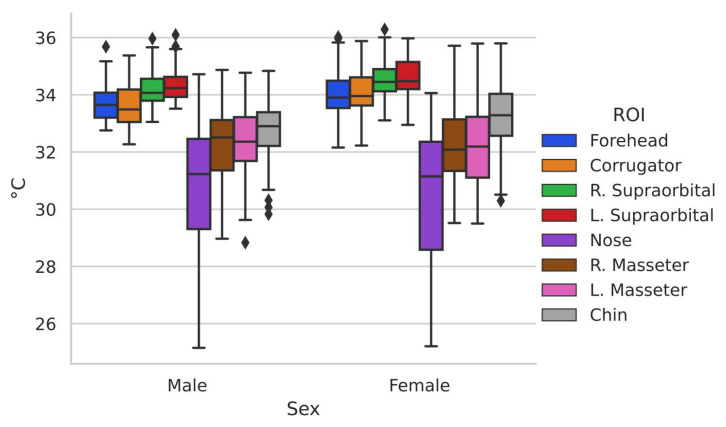
Box-and-whisker chart representing male and female gender.

To have a more favorable appreciation of the distribution of the data, it can be observed in Figure 4. This box-and-whisker plot shows the difference in temperature distribution between one group and another.

**Figure 5:**
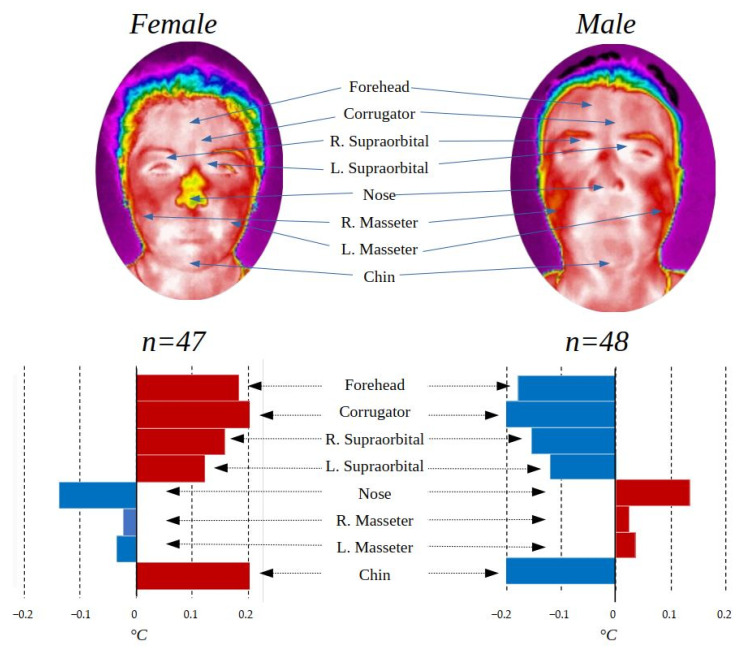
ROI comparison by gender.

It can be seen how even when there are differences in the distribution of the data in the two groups, they show similar behavior. To have a better appreciation of the differences in temperature in the basal state in the regions of interest between the two genders, Figure 5 is shown, graphically representing the behavior concerning the thermal distribution of the population in general.

The male gender group presented a higher temperature in the lower part of the face with exception of the chin while the female group in the upper part, while in the masseters, there are no outstanding differences. The differences found between genders are related to the physical composition of each one as addressed in [28], and this can also be seen in the face where it has been found that in general, men have a higher temperature than women [37]; statistical significance was found (*p* < 0.05) in facial temperature between genders for the upper part of the face (forehead, corrugator, and right supraorbital), but not for the other ROIs.

### 3.3. Age Comparison

In the same way that thermal differences can be found between genders, it is possible to find some differences according to age. In this research, there is a population with a wide range of ages, from 18 to 74 years old, so it was decided to divide it into two groups: under 25 years (*n* = 74) and over 25 years (*n* = 21). Table 4 shows the temperature averages and the standard deviation in each of the ROIs for the three age groups, as well as the *p*-values of the statistical test *t*-test for independent samples in the case of regions with normal distribution, and the Mann–Whitney u-test for those who did not present a normal distribution. Finally, the value of the effect size “r” is presented. The age comparison was made only with the control group to avoid possible alterations due to imbalance between groups.

To have a more beneficial appreciation of how the temperature data behave in the ROI, the box-and-whisker plots that represent the distribution of the data in each group are shown.

**Figure 6:**
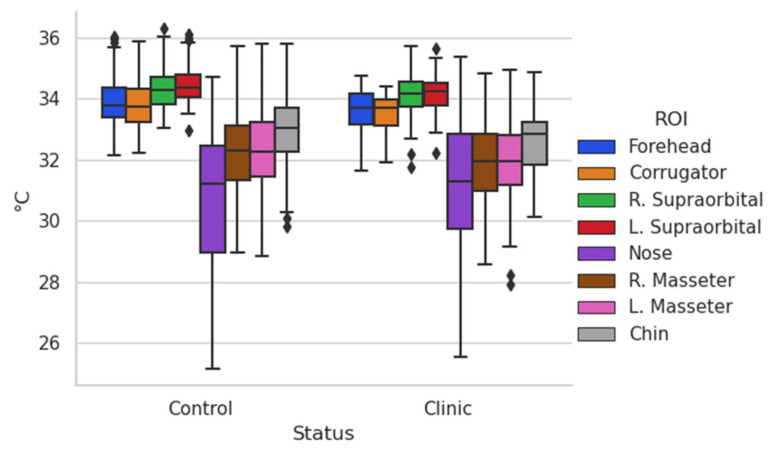
Box-and-whisker chart comparison by age.

The difference in distribution is notable between the groups, under 25 years old and over 25 years old, represented in Figure 6.

**Figure 7:**
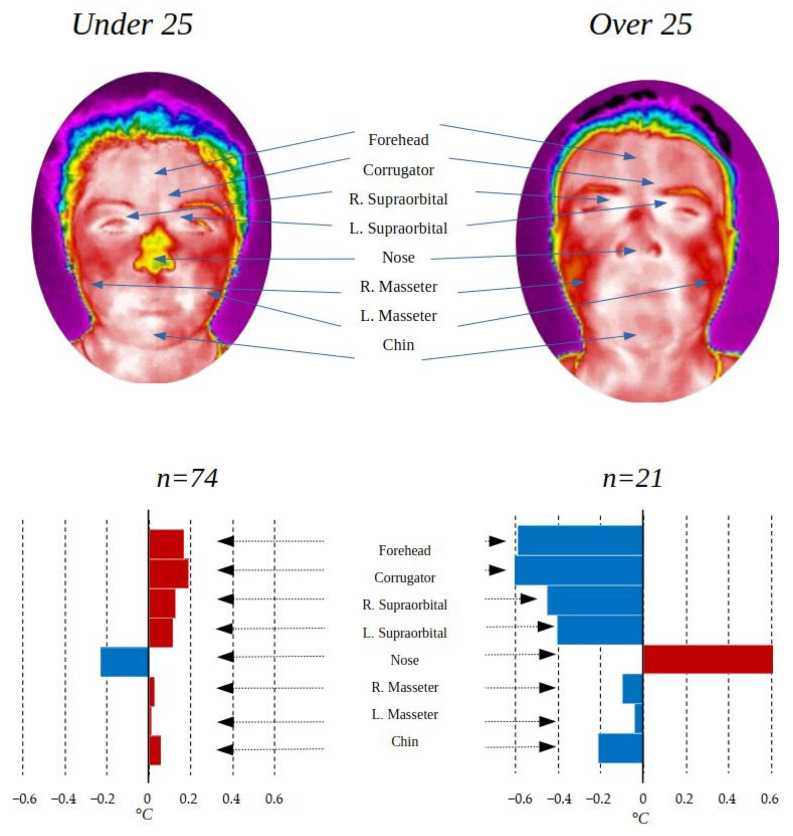
ROI comparison by age.

The difference in temperature by age in the ROI can be better appreciated in Figure 7.

The temperature differences found according to age are striking, with the greatest difference in the first 5 ROIs (forehead, corrugator, right supraorbital, left supraorbital, and nose). The difference in temperature with respect to age can be derived from the change in metabolism suffered as people age. As can be seen in Table 4, the changes between the group under 25 years of age and the rest are statistically significant in the 4 ROIs of the upper part of the face, presenting a *p*-value of less than 0.05, while the lower part is not.

Previous studies have not found significant differences according to age as presented [38], it is possible that the differentiation between different ages, or the selection of different ROIs are the cause of this change, or the fact that the population addressed in this study has a greater distribution between men and women. 

The differences found in this study may be related to the way in which the body adapts to an environment, since a younger person has a preferable body condition and with greater physical qualities, while an adult has more experience and better adaptation to different aspects of the environment, or it may even be related to the change in cognitive processes with age. Few studies have linked the increase in frontal temperature with cognitive processes identifying subgroups of people who respond distinctively to cognitive loads based on frontal thermal dominance right or left [39], in the study of types of attention [40] or, in emotions (with a strong association between asymmetry of the frontal EEG in those cases in which the affective task triggered a significant change in temperature [41]). For example, mindfulness practice has effects on the frontal region [42], suggesting that attention increases blood flow in this region. In addition, the authors of [43] reported that frontal temperature increased the more sophisticated a cognitive task was, while nasal temperature decreased. This could be due to the work overload of holding the corrugator muscle during a complex task. The Vascular Theory of Emotional Output [44] proposes that facial muscle movements modify the volume of air inhaled through the nose, which affects nasal and frontal temperature, which induces an affective response: the more fluid the respiratory cycle, the greater state of calm, of psychological well-being. On the other hand, respiratory distress can cause the person to feel unwell, fatigue, and increase frontal temperature. In a perceptive challenging situation, nasal temperature decreases as a result of rapid respiratory changes. It should be kept in mind that nasal temperature corresponds to cartilage. Moreover, the thick facial muscles (frontal, corrugator, orbicularis, chin) when activated by cognitive-affective processes require greater blood flow, increasing the temperature of that region. 

### 3.4. Clinic and Control Group Comparison

Having a population with the peculiarity of having individuals who were in hospital and others who were not, it was decided to separate into two groups, a control group with all the people who did not present diseases at the time of data acquisition (*n* = 95) and a clinical group with all those who were in some treatment (*n* = 57). The control group contains 48 male and 47 female participants, and 74 under 25 years old and 21 over that age. On other hand, the clinic group contains 4 male and 53 female participants, and 7 under 25 years old and 50 over that age.

The clinical group consisted of cancer survivors (more than 2 years from the time of cancer diagnosis), in addition to the inclusion criteria for the general study, specific criteria for the clinical group were taken into consideration, which were have a confirmed diagnosis and receive comprehensive medical treatment.

Table 5 shows the temperature averages and the standard deviation in the ROI addressed in this study for the two groups compared in this section, as well as the *p*-values of the statistical test *t*-test for independent samples in the case of the regions with normal distribution and the Mann–Whitney u-test for those that did not present normal distribution. Finally, the value of the effect size “r” is presented.

**Figure 8:**
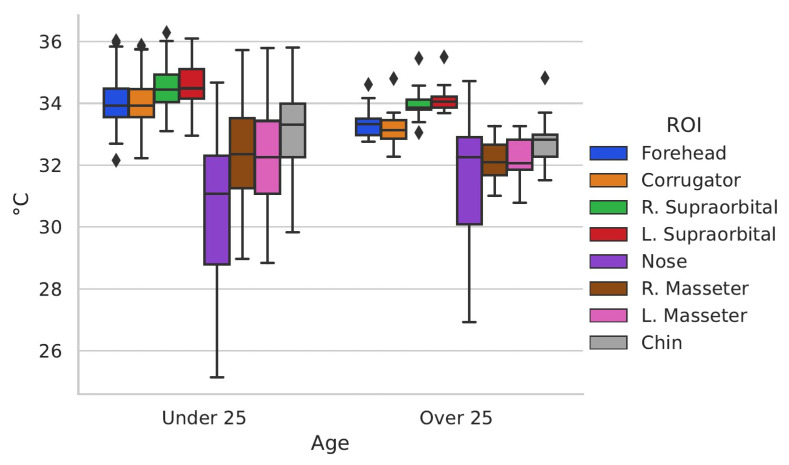
Box-and-whisker chart Control and Clinic group.

Figure 8 shows the box-and-whisker plots of the data from the control and clinical groups.

**Figure 9:**
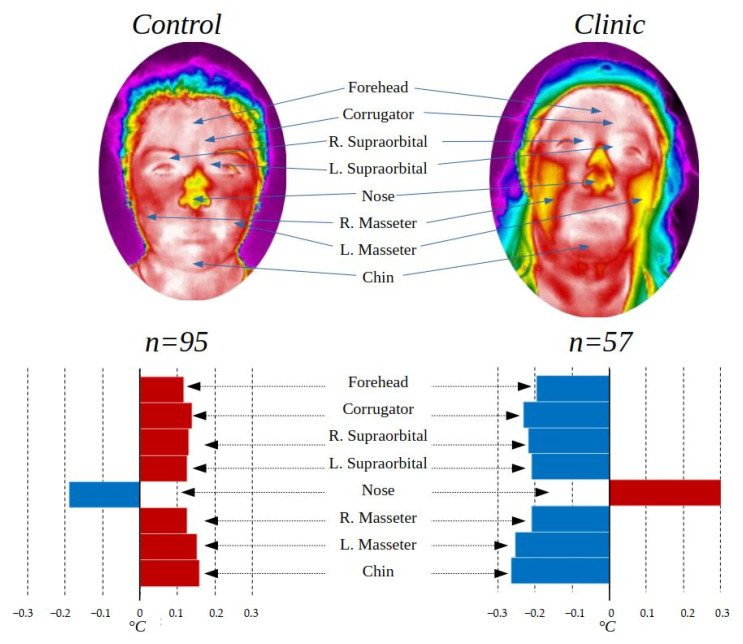
Control group and clinic group comparison.

Figure 8 shows the differences in data distribution between the control group and the clinical group. To have an enhanced appreciation of how the temperature varies according to these groups, Figure 9 was created.

The thermal variation shown in Figure 9 according to the clinical and control group shows that there is a higher temperature in the face of the control group except for the nose, which can be produced by the amounts of stress to which people who are fighting against a disease or even be symptoms of it, are subjected. Studies have shown that people hospitalized or facing diseases such as cancer present symptoms of high stress and anxiety [45,46], which is also observed in people with other diseases such as COVID-19 [47] and others [48,49,50].

The thermal differences between the control and clinical groups are statistically significant according to the *t*-test, obtaining a *p*-value < 0.05 in 4 of the 8 ROI evaluated.

## 4. Discussion

Research for the study of facial emotional response is traditionally performed with surface electromyography recording equipment, placing wired electrodes [34]. However, it has three drawbacks. First, the instrumentation limits spontaneous natural activity, the participant must remain with as little movement of his or her head as possible to reduce artifacts. Second, the time to implement (and remove the equipment, including storage of the equipment) is considerable (cleaning of the skin surface, placement of electrodes, recognition of the signal to the computer), which restricts its use for those recordings that have enough time. Third, regularly measuring surface electromyography, due to its nature, is only used in controlled environments, there are few portable pieces of recording equipment. Thermography represents an alternative to reduce the obstacles described above by allowing greater freedom of movement, limiting instrumentation times (it is enough for the participant to acclimatize for at least 5 min), and portability allows going to ecologically valid environments where they are developed for socio-affective activities to be examined. Even when studies have been published on the thermal behavior of different facial points [10,51,52], and some maps of the thermal regional distribution in the face in some clinical stances [38,53], as far as it is known, no standard of temperatures at facial ROIs in the basal state has been reported with which to compare clinical research: medical, dental, and psychophysiological. This would allow establishing parameters with which to compare if the temperature of a ROI is below, within, or above normal.

The temperature differences found in the groups addressed in this article are representative of a Mexican population and could vary depending on other regions of the world, in the same way, it is possible that some of the differences found in the clinical group against the control group are seen affected by the greater number of participants of one gender in one group than in the other.

As a prospective, it is planned to continue adding images and information to the thermal map in order to have the most suitable database regarding this topic in the future.

## 5. Conclusions

The creation of a face thermal map of the Mexican population in the basal state is of vital importance in order to continue and promote research within the clinical and psychological area, in addition to the possibility of being useful in other areas. The comparison between different population groups yields very interesting information, allowing some of the differences between them to be known; the contrast obtained between the clinical group and the control group is outstanding, being statistically significant (*p* < 0.05) in 4 of the 8 ROIs evaluated. Thermal differences were found in different ROIs between age groups, indicating that younger people have a warmer temperature. This is of utmost importance for thermal analysis derived from research since it could mean that biobehavioral functioning is different in this population, which is of utmost importance in psychophysiological and biomedical studies to establish the expected values between one and the other age group. Possible factors influencing this difference need to be examined.

## Figures and Tables

**Table 1:** Database resume.

Data	*n*	Average Age (Years)	SD
All	152	32.69	15.96
Male	52	24.21	5.88
Female	100	37.08	17.17
Under 25 yo	81	20.91	1.91
From 25 to 50 yo	36	33.97	7.79
Over 50 yo	35	58.57	6.30
Control group	95	22.67	3.88
Clinic group	57	49.35	14.47

SD is the standard deviation, *n* is the number of samples, and data is the type of data displayed (yo = years old).

**Table 2:** Thermal database averages.

ROI	Mean	SD	Normality Test
Forehead	33.80	0.76	***p* > 0.05**
Corrugator	33.70	0.80	***p* > 0.05**
Right Supraorbital	34.27	0.74	*p* = 0.01
Left Supraorbital	34.39	0.67	*p* = 0.00
Nose	30.82	2.39	*p* = 0.00
Right masseter	32.12	1.38	***p* > 0.05**
Left masseter	32.11	1.41	***p* > 0.05**
Chin	32.80	1.15	*p* = 0.00

SD is the standard deviation, mean is the average temperature in degrees centigrade at the selected point and, finally, ROI is the regions of interest (Bold values indicate *p* > 0.05).

**Table 3:** Gender comparison table.

ROI	Male		Female		*p*	*r*
	Mean	SD	Mean	SD		
Forehead	33.73	0.68	34.10	0.84	**0.02**	−0.23
Corrugator	33.58	0.73	34.10	0.88	**<0.01**	−0.30
Right Supraorbital	34.25	0.69	34.56	0.77	**0.04**	−0.20
Left Supraorbital	34.40	0.63	34.64	0.71	0.07	−0.18
Nose	30.77	2.42	30.50	2.47	0.59	0.05
Right masseter	32.27	1.35	32.22	1.46	0.87	0.01
Left masseter	32.29	1.29	32.22	1.48	0.80	0.02
Chin	32.74	1.13	33.18	1.21	0.07	−0.18

SD is the standard deviation, mean is the average temperature in degrees Celsius at the selected point, and, finally, ROI is the selected regions of interest (Bold values indicate *p* < 0.05).

**Table 4:** Age comparison table.

ROI	Under 25			Over 25		
	Mean	SD	*p*	*r*	Mean	SD
Forehead	34.08	0.78	**<0.01**	0.40	33.32	0.45
Corrugator	34.02	0.84	**<0.01**	0.41	33.18	0.50
Right Supraorbital	34.53	0.76	**<0.01**	0.32	33.95	0.49
Left Supraorbital	34.63	0.70	**<0.01**	0.31	34.11	0.39
Nose	30.40	2.47	0.08	−0.17	31.45	2.18
Right masseter	32.27	1.55	0.72	0.03	32.15	0.69
Left masseter	32.27	1.53	0.87	0.01	32.22	0.64
Chin	33.02	1.29	0.35	0.09	32.75	0.69

SD is the standard deviation, mean is the average temperature in degrees centigrade at the selected point, and, finally, ROI is the region of interest (Bold values indicate *p* < 0.05).

**Table 5:** Clinic and control group comparison table.

ROI	Control		Clinic		*p*	*r*
	Mean	SD	Mean	SD		
Forehead	33.91	0.78	33.60	0.68	**0.01**	**0.19**
Corrugator	33.84	0.85	33.46	0.66	**<0.01**	**0.22**
Right Supraorbital	34.40	0.75	34.05	0.69	0.06	−0.14
Left Supraorbital	34.52	0.67	34.18	0.61	**0.02**	**−0.18**
Nose	30.63	2.44	31.14	2.30	0.25	0.09
Right masseter	32.24	1.40	31.91	1.33	0.14	0.11
Left masseter	32.26	1.38	31.85	1.43	0.08	0.13
Chin	32.96	1.18	32.54	1.04	**0.02**	**−0.17**

SD is the standard deviation, mean is the average temperature in degrees centigrade at the selected point, and, finally, ROI is the region of interest (Bold values indicate *p* < 0.05).

## Data Availability

The data for this research can be found at the following link: https://data.mendeley.com/datasets/chhtccw6bm/draft?a=a7c526e9-e66c-4eb0-9831-8d9c397b6f53 (accessed on 13 September 2022).
